# Take-Home Video Shortens the Time to First Ambulation in Patients With Inguinal Hernia Repair Under General Anesthesia: A Retrospective Observational Study

**DOI:** 10.3389/fmed.2022.848280

**Published:** 2022-06-29

**Authors:** Guozhen Ma, Pengjun Jiang, Beirong Mo, Yijun Luo, Yongling Zhao, Xingguang Wang, Chunmiao Shi, Yanhui Huang

**Affiliations:** ^1^Day Surgery Care Unit, Huazhong University of Science and Technology Union Shenzhen Hospital, Shenzhen, China; ^2^School of Nursing, Philippine Women's University, Manila, Philippines; ^3^Department of Anorectal Surgery, Huazhong University of Science and Technology Union Shenzhen Hospital, Shenzhen, China; ^4^Department of Nursing, Huazhong University of Science and Technology Union Shenzhen Hospital, Shenzhen, China; ^5^Department of Anesthesiology, Huazhong University of Science and Technology Union Shenzhen Hospital, Shenzhen, China; ^6^Department of Gastrointestinal Surgery, Huazhong University of Science and Technology Union Shenzhen Hospital, Shenzhen, China

**Keywords:** take-home, video education, early ambulation, length of stay, general anesthesia, postoperative recovery

## Abstract

**Background:**

Data on the relationship between take-home video and the time to first ambulation remains scant. Here, we aimed to investigate whether viewed take-home video during pre-hospitalization is independently associated with the time to first ambulation in postoperative patients with inguinal hernia repair under general anesthesia.

**Methods:**

We retrospectively reviewed and analyzed the relationship between viewed take-home video and the time to first ambulation between September 2020 and October 2021.The independent *t*-tests or Mann-Whitney U-tests was used to compare the means of two groups (viewed take-home video and non-viewed take-home video). Chi-square test was used to compare the rates between the two groups. We used a linear regression model to see if there was a difference between exposure and outcome variable. Both models were used to observe the effect size of the exposed variable. Subgroup analysis was employed to assess the impact of various factors.

**Results:**

This study included a total of 120 patients with inguinal hernia repair under general anesthesia following day surgery. The average age of the participants in the two groups was 43.16 and 44.83 years, respectively, and about 82.5% of the patients were male. Our fully adjusted linear regression results showed that individuals in the viewed video group were associated with a decreased time to first ambulation (h) after adjusting for confounders (β = −0.50, 95%CI: −0.83, −0.17; *P* = 0.004). In addition, the linear regression analysis of the relationship between viewed video and length of stay showed that β = −2.10 (95%CI:CI: −3.85, −0.34; *P* = 0.021). Similarly, subgroup analysis yielded similar results for the viewed video group patients compared to those in the non-viewed video group.

**Conclusion:**

Taken together, our findings demonstrated that viewed video could shorten the time to first ambulation, which in turn reduce the length of stay in postoperative patients under general anesthesia.

## Introduction

2019 guidelines for day-case surgery point out the efforts to facilitate day surgery at the start of the millennium and recent drives to reduce length of stay and improve quality of recovery after surgery, which have ensured domestication of day surgery principles in modern patient care (1). The guidelines direct that early ambulation could achieve shorter hospitalization time and faster post-operative recovery (1). However, there is no standard definition of the time to first ambulation. Previous studies reported that the time to early ambulation may range from 4 h after surgery to 6 h post operation (2–4). Although these studies pointed out the the time horizon to early ambulation, they did not define occurrence of mobilization events (1, 4, 5).

An expert consensus on standard process of ambulatory surgery for inguinal hernia states that health education can reduce the psychological stress response caused by surgical operations to patients, shorten the hospital stay, save medical expenses, and relieve patient anxiety and postoperative pain (6). Health education is an efficient tool to accomplish shorter stay time and faster postoperative recovery. Previous data demonstrated the use and effects of videos in health education in many fields (7–9). However, data on the effect of video education on the time to early ambulation in patients with inguinal hernia following day surgery remain scanty. This study aimed to analyze the relationship between viewed take-home video and the time to first ambulation.

Inguinal hernia is a common and frequently occurring condition in surgery, and performance of inguinal hernia day surgery has considerable socio-economic value (10). The use of intervention methods such as take-home video, which is formulated *via* a multidisciplinary team (MDT), adopted the theoretical frame for knowledge, attitude, and practice (KAP), and used the first ambulation system of recovery skills covered in post anesthesia discharge scoring system (PADSS), does not only shorten the time to first ambulation, reduce the length of hospital stay and early recovery, but also improves the efficiency of nurses, and provides a reference for the medical staff to guide the time to first ambulation.

## Methods

### Study Design

This retrospective study was conducted from September 2020 to October 2021 in a tertiary hospital named Shenzhen Sixth People's Hospital. When making the appointment for patients' operation at 3–5 days before surgery, medical staff at the day surgery management center conducted face-to-face perioperative care education as well as all patients were given the same educational brochure and QR code of take-home video for perioperative early recovery knowledge and skills instruction. During the pre-hospitalization period, patients could choose to read the health booklet or view the take-home video by scanning the QR code. For patients who chose to view the video, and at the end of the video, they had to fill in their name, the day of the proposed surgery and click “watched” to end the video, then the completed information was automatically sent to the Questionnaire Star (Changsha Ranxing Information Technology Co., Ltd, a professional online questionnaire, and then automatically generated the data on whether to watch the video).

All anesthesia and surgeries (laparoscopy) were performed by the same anesthesiologist and specialist doctor. The mode of anesthesia included general anesthesia (propofol, remifentanil, muscle relaxants and NSAIDs) combined with preoperative nerve block (quadratus lumborum block). In addition, postoperative pain was relieved using diclofenac sodium which was orally administrated for 5 consecutive days.

### Ethics Approval

Data on patients diagnosed with inguinal hernia was obtained from the day surgery care unit of the Shenzhen Nanshan Sixth People's Hospital, Shenzhen, China. The identities of participants were concealed to safeguard the patients' privacy. Besides, the information was abstracted from the electronic data acquisition system. Since this study was conducted retrospectively, ethical approval was waived by the local Ethics Committee of the Shenzhen Nanshan Sixth People's Hospital, and all the procedures were part of the routine care.

### Study Population

In this study, since data collection was retrospective, we included only a specific team by setting nadir criteria in order to avoid the possible influence of different teams on the results.

**Figure 1:**
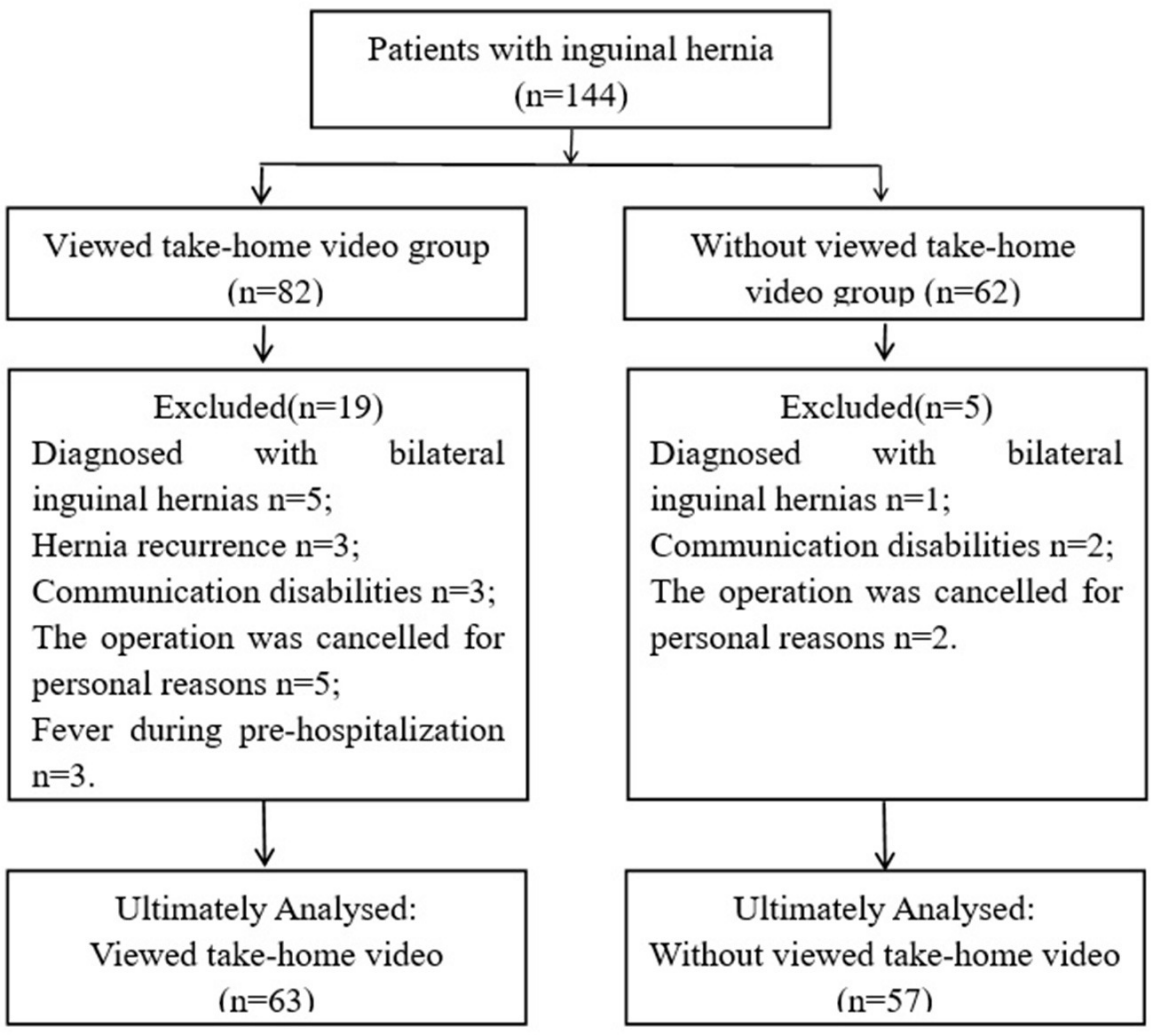
Study flow diagram.

A total of 144 patients with laparoscopic inguinal hernia repair between September 2020 and October 2021 were retrospectively screened. Twenty-four patients were excluded because they were diagnosed with bilateral inguinal hernias and hernia recurrence, communication disabilities, personal reasons or fever during pre-hospitalization. Thereafter, 120 patients were included and then divided into viewed take-home video (*n* = 63) and non-viewed take-home video (*n* = 57) groups (Figure 1).

We included patients who were diagnosed with unilateral inguinal hernias and had herniorrhaphy for the first time, those who were under grade I-II of the American Association of Anesthesiologists (ASA) (11) and those with normal communication skills. In addition, patients with normal preoperative vital signs and those who viewed the take-home video in full were also included in the study. However, patients with general contraindications such as a fever, anticoagulant drug use, significant co-morbidities, poor general conditions, and patients who were younger than 18 years were excluded from the study. Besides, those with contraindications to anesthesia (6) and poor adherence to view video as well as complications such as mental disorders were also excluded.

### Independent Variable

**Figure 2:**
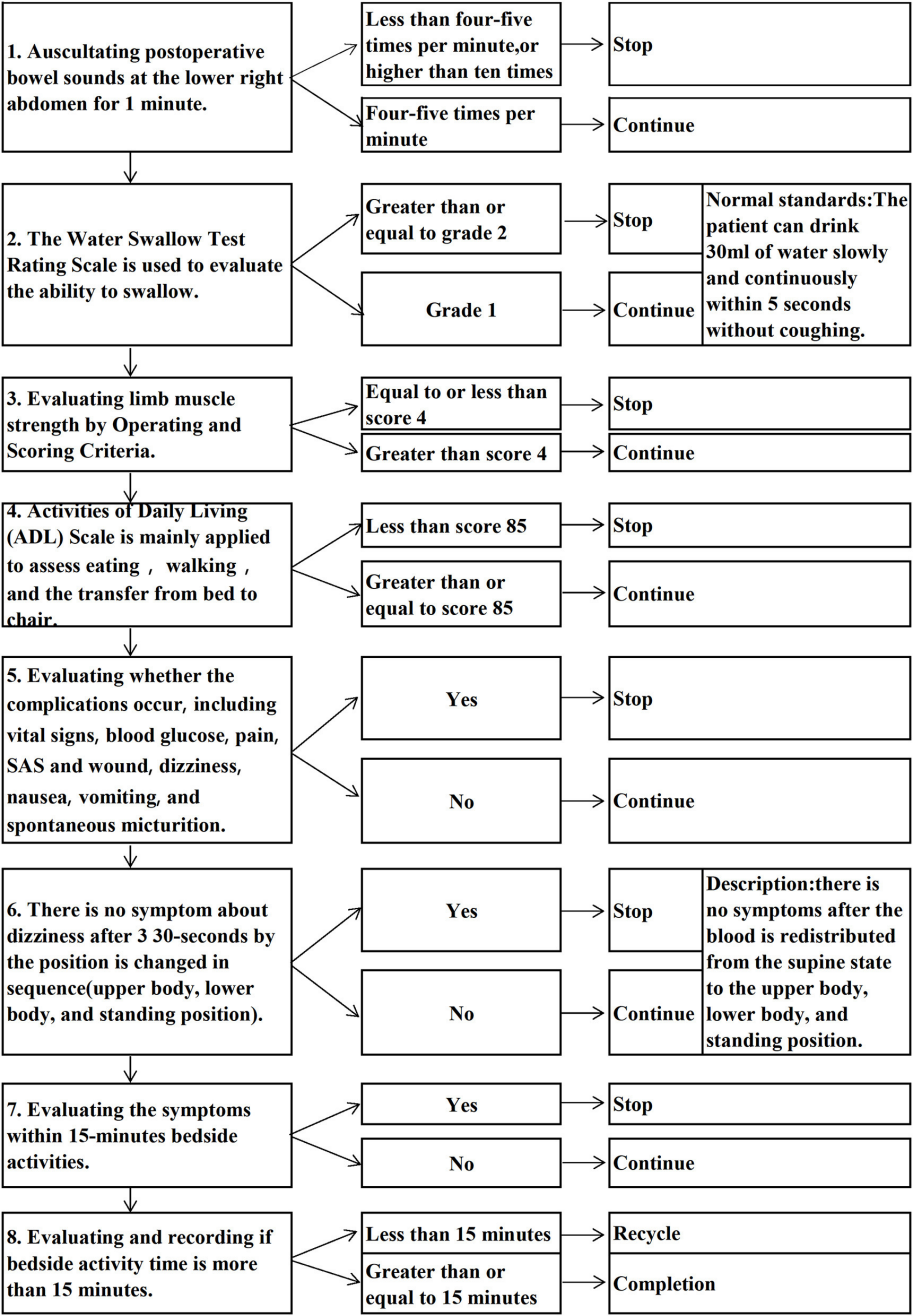
The workflow of the first ambulation.

The independent variable (categorical variable) was viewed take-home video, which was recorded by a MDT who wrote the script and role played. In addition, the contents of the take-home video were formulated based on the guidelines and clinical practices (6) and the theoretical frame for KAP (12).The take-home video consisted of three parts, namely Knowledge: Public science education to disease knowledge, surgical procedure, and postoperative recovery by surgeon in charge (4 min);Health education on perioperative care and precautions by nurses in charge of the day surgery ward (3min);Attitude:Peer support education (13) interview about perioperative experiences of post-operative patients served at our ward (5 min); Practice: Selected corresponding skills of accelerated postoperative recovery by deciphering PADSS (14) and measurement and observation of vital signs, a skill of first ambulation after surgery (Figure 2), assessment and observation of postoperative complication as well as management and evaluation of wound pain (7 min). The patients were required to view the take-home video at pre-hospitalization and the total time of video playback was 19 min (Supplementary Video).

### Dependent Variable

The outcome variable (continuous variable) was obtained following the previously published guidelines and studies (1, 4, 5, 15, 16). In addition, the time to first ambulation was measured as follows: a registration form for the time to first mobilization was distributed upon admission to the hospital and the patient would walk for at least 15 min after getting out of bed for the first time (3, 17). Length of stay was defined as number of hours of stay from admission to discharge (18).

### Covariates

Demographic variables, patients' satisfaction (19), self-rating anxiety scale (SAS) (20), pain scores (21), and time to first dietary were included as covariates (Supplementary Covariates).

### Statistical Analysis

The independent variable for this study was whether viewed take-home video or not (dichotomous variable, 1 = yes, 0 = no). The primary outcome variables were the time to first ambulation as well as length of stay. Therefore, we first observed the distribution of baseline data of participants was whether statistically different between the viewed take-home video and non-viewed take-home video groups. Among them, the independent *t*-tests (normal distribution) or Mann-Whitney U-tests (skewed distribution) were used to compare the means of two groups, Chi-square test was used to compare the rates between the two groups. Furthermore, given that the outcome variables were continuous, we used a linear regression model to see if there was a difference in time to first ambulation and length of stay in the viewed take-home video group compared to the non-viewed take-home video group, and what the difference was, and reported the effect values β and their confidence intervals.

To control for confounders, we used a multivariable model. To observe whether the effect values were stable, we presented both the fully-adjusted model with the unadjusted model and after controlling for confounders. To assess whether the effect values can be applied to different populations, we used indicators such as gender as stratified variables to observe whether the trend of effect values and credible intervals of exposure variables and outcome variables were stable at different stratification.

All statistical analyses were done using Empower Stats (version 3.4.3). Packages used: ggplot, car, caret and corrplot.

## Results

### Baseline Characteristics of the Selected Participants

We analyzed the baseline characteristics of the selected patients as shown in Table 1. The average age of the participants in the viewed take-home video and non-viewed take-home video group was 43.16 and 44.83 years, respectively. About 82.5% of the patients were male. In addition, our results showed that there was no significant difference in age, gender, body mass index (BMI), education level, pain score, and hospital cost between the two groups (all the *p*-values were ≥ 0.05). However, the viewed video group had significant differences in the time to first ambulation, length of stay, patient satisfaction, SAS, and time to first dietary (all the *p* < 0.05).

**Table 1:** Baseline characteristics of participants [*n* (%)/ mean ± SD].

**Variable**	**Non-viewed take-home** **video (*n* = 57)**	**Viewed take-home** **video (*n* = 63)**	***P***
**Age (years)**	44.83 ± 12.97	43.16 ± 14.09	0.354
**Gender (** ***n*** **, %)**			0.091
Female	6 (10.53%)	15 (23.81%)	
Male	51 (89.47%)	48 (76.19%)	
**BMI(kg/m** ^**2**^ **)**	22.17 ± 2.57	22.99 ± 3.23	0.132
**Education level** **(*****n*****, %)**			0.591
Primary school and under	2 (3.51%)	1 (1.59%)	
Middle school	4 (7.02%)	7 (11.11%)	
High school	10 (17.54%)	11 (17.46%)	
College and above	41 (71.93%)	44 (69.84%)	
**Time to first ambulation** (h, mean ± SD)	3.73 ± 0.75	3.34 ± 0.72	0.005
**Length of stay** (h, mean ± SD)	11.97 ± 4.11	10.31 ± 3.07	0.013
**Patient satisfaction** (scores, mean ± SD)	94.98 ± 2.18	96.35 ± 2.60	0.002
**Self-rating anxiety scale** (scores, mean ± SD)	45.91 ± 6.19	42.48 ± 5.96	0.002
**Pain scores** (*n*, %)			0.781
<4 scores	51 (89.47%)	55 (87.30%)	
≥4 scores	6 (10.53%)	8 (12.70%)	
**Time to first dietary** (h, mean ± SD)	3.34 ± 0.81	3.00 ± 0.81	0.023
**Hospital costs** (yuan, mean ± SD)	12,360.39 ± 757.74	11,969.17 ± 1,286.91	0.048

### Subgroup Analysis

We then used covariates as the stratification variables to assess the trend of effect sizes in the time to first ambulation and length of stay (Table 2). The effect size of viewed video on the time to first ambulation was required to be interpreted with caution due to the wide confidence interval and smaller female sample (including effect size for length of stay), ≥65 years in age stratification (22), and middle school subgroup. No statistical tests were performed due to small primary school and under sample size.

**Table 2:** Effect size of viewed take-home video on the time to first ambulation (h) and length of stay (h) in prespecified and exploratory subgroups in each subgroup.

**Covariates**	**N**	**Time to first ambulation**		**Length of stay**	
		**β (95%CI)**	***P***	**β (95%CI)**	***P***
**Gender**					
Female	21	−0.91 (−1.61, −0.20)	0.021	−2.80 (−4.66, −0.95)	0.008
Male	99	−0.39 (−0.66, −0.12)	0.006	−1.54 (−3.06, −0.01)	0.051
**Age (years)**					
≤ 40	50	−0.41 (−0.90, 0.07)	0.102	−1.41 (−3.32,0.51)	0.156
41–64	57	−0.31 (−0.65, 0.03)	0.076	−1.84 (−3.91, 0.24)	0.089
≥65	13	−0.86 (−1.39, −0.33)	0.009	−2.07 (−5.12, 0.98)	0.210
**BMI(kg/m** ^**2**^ **)**					
<18.5	39	−0.32 (−0.82, 0.19)	0.226	−2.48 (−4.60, −0.35)	0.029
18.5–24.9	41	−0.36 (−0.86, 0.14)	0.167	−1.51 (−3.58, 0.55)	0.159
25–30	40	−0.42 (−0.78, −0.06)	0.026	−1.02 (−3.65, 1.61)	0.453
**Education level**					
Primary school and under	4	Refrence (0)		Refrence (0)	
Middle school	11	−0.77 (−1.41, −0.13)	0.042	−2.46 (−6.23, 1.30)	0.232
High school	21	−0.28 (−0.88, 0.32)	0.371	−1.60 (−3.69, 0.50)	0.152
College and above	84	−0.33 (−0.66, 0.01)	0.058	−1.69 (−3.38, 0.01)	0.055
**Patients satisfaction (scores)**					
<95	21	−0.14 (−0.94, 0.66)	0.735	0.39 (−2.60, 3.38)	0.800
≥95	99	−0.43 (−0.71, −0.16)	0.002	−2.09 (−3.52, −0.66)	0.005
**Self-rating anxiety sca**l**e (scores)**					
<50	92	−0.43 (−0.74, −0.12)	0.007	−1.89 (−3.42, −0.36)	0.018
≥50	28	−0.29 (−0.86, 0.29)	0.340	−1.52 (−4.18, 1.14)	0.274
**Pain scores (scores)**					
<4	106	−0.34 (−0.62, −0.07)	0.014	−1.60 (−3.04, −0.17)	0.031
≥4	14	−0.78 (−167, 0.10)	0.109	−2.06 (−4.38, 0.25)	0.106

The analysis showed that there were significant differences in patients' BMI (≤ 30 kg/m^2^, <18.5 kg/m^2^), satisfaction (≥95 score), SAS (<50 score), and pain scores (<4 score) with respect to the interactions of the time to first ambulation and the length of stay. Besides, the interactions between gender (male) and the time to first ambulation were statistically significant (β = −0.39; 95%CI: −0.66, −0.12; *P* = 0.006). The effects of BMI (≤ 30 kg/m^2^), patients' satisfaction (≥95 score), SAS (<50 score), and pain scores (<4 score) on the time to first ambulation were 25.2 min (β = −0.42; 95%CI: −0.78, −0.06; *P* = 0.026), 25.8min (β = −0.43;95%CI: −0.71, −0.16; *P* = *0.002*), 25.8 min (β = −0.43; 95%CI: −0.74, −0.12; *P* = *0.007*), and 20.4 min (β = −0.34; 95%CI: −0.62, −0.07; *P* = *0.014*), respectively. In addition, the effects of BMI (<18.5 kg/m^2^), patients' satisfaction (≥95 score), SAS (<50 score), and pain scores (<4 score) on the hospital stay were 148.8 min (β = −2.48; 95%CI: −4.60, −0.35; *P* = 0.029),125.4min (β = −2.09; 95%CI: −3.52, −0.66; *P* = *0.005*), 113.4 min (β = −1.89; 95%CI: −3.42, −0.36; *P* = 0.018), and 96 min (β = −1.60; 95%CI: −3.04, −0.17; *P* = 0.031) minutes, respectively.

### Multivariate Liner Regression Analysis

We constructed 2 models to analyze the independent effects of exposure variable on the time to first ambulation and length of stay. We defined the effect sizes (β) and 95% confidence intervals as shown in Table 3.

**Table 3:** Relationship between exposure variable and the time to first ambulation and length of stay(h).

**Exposure variable**	**Time to first ambulation**				**Length of stay**			
	**Crude mode**	***P***	**Adjusted mode**	***P***	**Crude mode**	***P***	**Adjusted mode**	***P***
	**β (95%CI)**		**β (95%CI)**		**β (95%CI)**		**β (95%CI)**	
Viewed take-home video	−0.39 (−0.65, −0.12)	0.005	−0.50 (−0.83, −0.17)	0.004	−1.66 (−2.95, −0.37)	0.013	−2.10 (−3.85, −0.34)	0.021
Age	−0.00 (−0.01, 0.01)	0.458	−0.01 (−0.03, 0.01)	0.284	−0.03 (−0.08, 0.02)	0.227	−0.01 (−0.10, 0.08)	0.884
Female	Refrence (0)		Refrence (0)		Refrence (0)		Refrence (0)	
Male	−0.48 (−0.83, −0.13)	0.008	−0.54 (−1.04, −0.05)	0.035	−0.06 (−1.80, 1.68)	0.947	−0.85 (−3.41, 1.72)	0.519
BMI <18.5 kg/m^2^	Refrence (0)		Refrence (0)		Refrence (0)		Refrence (0)	
BMI:18.5-24.9 kg/m^2^	0.03 (−0.30, 0.36)	0.852	0.13 (−0.23, 0.50)	0.468	−0.46 (−2.09, 1.16)	0.577	−0.14 (−2.03, 1.76)	0.889
BMI:25–30 kg/m^2^	−0.29 (−0.61, 0.04)	0.092	−0.23 (−0.60,0.13)	0.208	−0.21 (−1.84, 1.43)	0.806	−0.16 (−2.07, 1.74)	0.868
Education level	−0.00 (−0.17, 0.17)	0.998	−0.26 (−0.55, 0.03)	0.077	0.66 (−0.17, 1.48)	0.121	0.42 (−1.09, 1.93)	0.588
SAS	−0.00 (−0.02, 0.02)	0.923	−0.00 (−0.02, 0.02)	0.970	−0.02 (−0.12, 0.09)	0757	−0.01 (−0.12, 0.11)	0.923
Pain scores	0.08 (−0.07, 0.24)	0.275	−0.07 (−0.26, 0.12)	0.482	−0.39 (−1.12, 0.35)	0.307	−0.41 (−1.39, 0.57)	0.411
Time to first dietary	0.09 (−0.08, 0.25)	0.305	0.09 (−0.08, 0.26)	0.319	−0.14 (−0.95, 0.66)	0.726	−0.04 (−0.90, 0.82)	0.931

*Crude model was adjusted for none. Adjusted model was adjusted for: Age; Gender; BMI; Education level; SAS; Patients satisfaction; Pain scores; Time to first dietary*.

Of all the exposure factors, only viewed take-home video had a statistically significant difference in the effect of the two outcome variables. Of these, the male exposure factor, only had a statistically significant effect on the time to first ambulation (the outcome variable). No statistical tests were performed due to small female sample size.

In the unadjusted model (Model I), the effect of viewed video on the time to first ambulation and length of stay was decreased by 23.4 min (β = −0.39, 95%CI: −0.65, −0.12; *P* = 0.005) and 99.6 min (β = −1.66, 95%CI: −2.95, −0.37; *P* = 0.013), respectively. In model II, the fully-adjusted linear regression analysis showed that individuals in the viewed video group were associated with a decreased time to first ambulation after adjustment for confounders (β = −0.50, 95%CI: −0.83, −0.17; *P* = 0.004). In addition, the relationships with viewed video and length of stay were analyzed and showed a β = −2.10 (95%CI: −3.85, −0.34; *P* = 0.021).

In the two models (I and II), the effect of the male on the time to first ambulation was decreased by 28.8 min (β = −0.48, 95%CI: −0.83, −0.13; *P* = 0.008) and 32.4 min (β = −0.54, 95%CI: −1.04, −0.05; *P* = 0.035), respectively.

## Discussion

### Skills for First Ambulation in the Take-Home Video

How to continuously improve the quality of service under ensuring patient safety and reduced hospital stay, as previously shown, early mobility can lead to accelerated recovery and a shortened hospital stay, which is a primary element for a rapid recovery plan (23). By standardizing the skills for first ambulation in the videos, the patients would achieve early ambulation, and improve hospitalization experience.

The workflow for the first ambulation includes 8 steps, and requires the patient to be able to eat, move, and have no discomfort, then to ambulate after singing the trilogy. However, for the safety of the patients, nurses are required to evaluate bowel sounds and ability to swallow before eating, evaluate limb muscle strength and ADL (Activities of Daily Living) before moving, evaluate the occurrence of complications such as vital signs, blood glucose, pain, SAS and wound, dizziness, nausea, vomiting, and spontaneous micturition. Besides, singing the trilogy shows that there is no dizziness holding per position 30-s by the position is changed in sequence (upper body, lower body, and standing position). In clinical practice, through the timely assessments by the nurses coupled with the patients' cooperation, our analysis showed that the time to first ambulation, length of stay, and the time to first dietary of the viewed take-home video group were lower compared to the no-view take-home video group. Because the skills of first ambulation after surgery in the take-home video included the contents of entries in PADSS and ≥9 points (24), our patients were discharged with a doctor's recommendation within 10 min after first ambulation (equal to the postoperative discharge time). In contrast, Jaensson et al. (25) reported that 82% of patients undergoing day surgery were discharged <270 min following the procedures, our time to first ambulation after surgery was 3.34 ± 0.72 h.

Because the patients' payment method was a packaged charge for medical insurance, there was no statistical difference in the hospital costs for the two groups of patients.

To better analyze the impact of take-home video on the time to first ambulation and length of stay, we used a linear regression model, adjusted for potential confounding factors, to perform sensitivity analyses. Linear regression indicated that differences of viewed take-home video and male were statistically significant when controlling for age, BMI, education level, SAS, patient satisfaction, pain score, time to first dietary (Table 3). Of these, the results of fully-adjusted linear regression showed that patients in the viewed video group were associated with a decreased by 30 min for time to first ambulation (β = −0.50, 95%CI: −0.83, −0.17) and by 126 min for length of stay (β = −2.10, 95%CI: −3.85, −0.34). According to the standard of 300 patients with inguinal hernia, which states that the time for each patient to early ambulation is 30 min and the reduced hospitalization time is 126 min, the total time for 300 patients to early ambulation is 150 h and reduced length of stay is 630 h. Thus, the above reduced service time demonstrated that the protocol not only improves the work efficiency of the staff and the operating efficiency, but also saves the cost of patient care as well as corresponding medical resources, etc. In linear regression model analysis, there was a statistically significant difference in the effect of males on the time to first ambulation, but not on length of stay. We will continue to increase the sample size to observe the effect of different genders on the length of stay after viewed take-home video to provide a reference for future intensive interventions depending on gender.

In addition, subgroup analysis was used to better understand the association between the viewed take-home video and the first ambulation and length of stay in different populations. The data showed that the interactions between gender and first ambulation were statistically significant. This may be related to Shenzhen city's fast-paced lifestyle as well as the idea that early return to work after surgery. Our observations are consistent with results by Blockhaus et al. females had a higher adherence with the health education (26), however, the effect size of viewed video on the time to first ambulation was required to be interpreted with caution due to the wide confidence interval and smaller female sample. Eddib et al. found that BMI did not correlate with procedure duration and length of stay (27), however, our data showed that the impact on length of stay for day surgery patients at a BMI <18.5 kg/m^2^ was a reduction of 2.48 h (β = −2.48; 95% CI: −4.60, −0.35). Data from the Mudumbai et al. study showed that overweight patients feared exercise and avoided physical activity (28), but our observational data showed that patients with a BMI (25–30) were 25.2 min (β = −0.42; 95%CI: −0.78, −0.06) earlier in first ambulation after surgery. For this, more studies involving larger sample size and more medical centers are needed to explore the association between BMI and first ambulation. According to the satisfaction management requirements of the hospital, SAS and pain scoring standards, we analyzed the data by layers (19–21). There were significant differences in the patients' satisfaction (≥95 score), SAS (<50 score), and pain scores (<4 score), with respect to the interactions of the time to first ambulation and the length of stay. Thus, in clinical work, enhancing patient satisfaction, psychological care and pain management could facilitate the patients' postoperative recovery and reduce the length of hospital stay.

We, for the first time, examined the independent association between viewed take-home video and the time to first ambulation in patients with inguinal hernia following day surgery. Therefore, our findings would provide useful insights on the establishment of predictive models for the time to first ambulation.

### Provision of a Reference Basis for Accurate Service Time

Patient-centered health services is valuable and should be central to any transformative strategy management effort (29). Although we have moved into a new era of precision medicine, there is no corresponding “precision service.” We aimed to clarify the time to first ambulation after various surgery, and provide a reference basis for ward nurses to encourage patients to early ambulation.

At present, strategies to improve the first ambulation are largely dependent on nurses. However, due to their busy working schedules, nurses mainly encourage early ambulation and have less personally assistance and supervision to the first mobilization, lack continuous assessment and evaluation to early ambulation (16). Early ambulation is an essential element of accelerated recovery (23). However, the current guidance time for accelerated recovery of the patients undergoing inguinal hernia surgery is inconsistent (30, 31). In the nursing practice, the ward and anesthesia nurse hand over the patient after the return to the ward from the post-anesthesia care unit (PACU), after that, there is assessment prior to first ambulation. Patients who eat, move, and have no discomfort could ambulate after singing the trilogy. Our analysis showed that the time to first ambulation was faster compared to previous studies (25, 30, 31). Thus, our data may be used as a reference standard for the time to first ambulation.

It is recommended that the first ambulation be modeled into an operational criterion to guide clinical nursing. For example, the workflow specified the time for the first ambulation after the patient is admitted to the hospital, the nurses would be sent out for bedside recovery supervision after patient admission to record completion time to first ambulation, the content of the assessment and corresponding skills before the first ambulation, and the requirements for doctor-patient cooperation. In addition, the nursing behaviors are specified by standardized work procedures, to ensure the nurses consciously and actively intervene in the patient's first ambulation, for early recovery.

### Advantages of Take-Home Video

To improve compliance of the patients undergoing day surgery with the recovery skills in the take-home video, we employed the theoretical frame for KAP. The video content was designed through a wisdom-of-crowd of the MDT, and the attending surgeon recorded the knowledge of disease popularization and education, which not only increased the patients' knowledge of diseases and surgery, but also increased the professional trust of the doctor.

Patients who were treated for the same disease in our ward were invited to conduct peer support education. This did not only increase the trust of the patients in the surgical experience but also increased their confidence in the capabilities of the medical staff. Consistent with the findings of Cherrington et al. (13), the former patients' intervention effectively reduced the preoperative psychological pressure of the current patients. The SAS score of the viewed take-home video group was lower than that of the without viewed take-home video group.

The skill system for the first ambulation in the take-home video was extracted from the PADSS, where the patients' were discharged when the PADSS ≥9 scores (14). Thus, when the patient successfully performed the skills of first ambulation, it also indicated that the patient has reached the discharge standard.

In addition, the patients learned about the disease and surgery by viewing the video. Through peer education, the patients saw the effect of peer's procedure and felt the experience of peer's operation. This method made the patients to have confidence in our service capabilities and improved their compliance with our work requirements. For example, taking painkillers on time after surgery to prevent pain, and cooperating with recovery procedures according to the requirements of the first ambulation were implemented. Our analysis demonstrated that viewing the video improved the patient's satisfaction and reduced the preoperative psychological pressure compared with those who did not view the video. In addition, although the pain scores for the two groups were not statistically significant, more than 87% of the patients in the two groups had pain scores ≤ 3.

### Strengths and Limitations

This study uses the practical perspective on PADSS (14) as theoretical underpinning. Rigorously designed system of skills for first ambulation. Not only does it ensure patient safety, it also advances the patient's first ambulation, which in turn reduces the length of stay.

It is regrettable that we have not yet completed the statistical analysis of the corresponding data on fluids amounts during surgery, operation time, operative bleeding volume and so on, so this part of the data is not complete. An additional limitation is that this study did not report postdischarge complications. Third, our study only included surgical data from a specific team. However, the next paper will report those question. Therefore, there was a certain deficiency in the universality and extrapolation of our findings, its extrapolation needs to be interpreted more carefully.

## Conclusion

Taken together, our study showed that viewed take-home videos during the pre-hospitalization were significantly associated with the time to first ambulation and the viewed take-home video could decrease the postoperative time to first ambulation in patients under general anesthesia following day surgery. We, therefore, recommend routine viewing of take-home video during pre-hospitalization to shorten the time to first ambulation, which in turn reduce the length of stay in postoperative patients under general anesthesia.

## Data Availability Statement

The original contributions presented in the study are included in the article/Supplementary Material, further inquiries can be directed to the corresponding author.

## Author Contributions

GM and BM contributed to the conception and design of the study. GM and PJ performed the acquisition, statistical analysis, and were the major contributors in writing the first draft of the manuscript. All authors contributed to the article and approved the submitted version.

## Funding

This study was supported by the Key Project of Science and Technology Plan of Nanshan District, Shenzhen City (No. 2020023).

## Conflict of Interest

The authors declare that the research was conducted in the absence of any commercial or financial relationships that could be construed as a potential conflict of interest.

## Publisher's Note

All claims expressed in this article are solely those of the authors and do not necessarily represent those of their affiliated organizations, or those of the publisher, the editors and the reviewers. Any product that may be evaluated in this article, or claim that may be made by its manufacturer, is not guaranteed or endorsed by the publisher.
