# Supplementary material for: Take-Home Video Shortens the Time to First Ambulation in Patients With Inguinal Hernia Repair Under General Anesthesia: A Retrospective Observational Study
Source: Front Med (Lausanne). 2022 Jun 29;9:848280. doi: 10.3389/fmed.2022.848280 (PMC9278018; doi:10.3389/fmed.2022.848280)
Supplement: Supplementary file 4 [file Table_1.DOC]

**The Evaluation Criteria of the Covariates**

**1.Self-rating Anxiety Scale (SAS)**

SAS is used to evaluate the mental state of the patients before the operation. The scale consists of 20 items, grades 1–4 are used to add up the scores, and the standard scores calculated. The critical criterion score is 50, mild anxiety score is 50–59, moderate anxiety score is 60–69, and severe anxiety score is ≥70.

The content validity index of the scale is 0.924, and Cronbach’s coefficient is 0.708(1).

**2.Pain Numerical Rating Scale (****NRS)**

A patient uses 0–10 scale to describe the pain intensity, 0 means no pain, while 10 means maximum pain. The higher the number, the more the pain. NRS is not limited by age and educational background. The elderly and individuals with low academic qualifications could be subjected to the assessment similarly, and strikingly, the evaluation accuracy is higher. For people with impaired vision, nurses are allowed to dictate the evaluation (2).

**3.Questionnaire of Satisfaction**

For the questionnaire of satisfaction, the content validity was 0.85, and the Cronbach’s α coefficient was 0.91.

With a total score of 100 points, the questionnaire adopts 5-levels: 1 is very dissatisfied (20 points); 2 is relatively dissatisfied (40 points); 3 is generally satisfied (60 points); 4 is relatively satisfied (80 points); 5 is very satisfied (100 points).

The contents of the questionnaire are composed of service attitude, basic nursing, nursing education, ward management, and working ability.

The questionnaire consists of 13 items. According to the goal of the hospital’s satisfaction plan at the present stage, the score is 91–100 for very satisfied, 81–90 for basic satisfaction, and ≤80 for dissatisfaction(3).

**References**

1.Liu CL, Liu L, Zhang Y, Dai XZ, Wu H(2017) Prevalence and its associated

psychological variables of symptoms of depression and anxiety among ovarian cancer patients in China: a cross-sectional study. Health Qual Life Outcomes15(1):161.

<https://doi.org/10.1186/s12955-017-0738-1.>

2.Jang JH, Park WH, Kim HI, Chang SO(2019)Ways of reasoning used by nurses in

postoperative pain assessment.Pain Manag Nurs S1524-9042(18):30031-6. <https://doi.org/10.1016/j.pmn.2019.09.008.>

3.Ma GZ, Mo BR, Jiang Pj, Shen Hl,Deng JM(2017).Effect of health education

improvement on promotion of timely vaccination among migrant children in community.Chinese Journal of Nursing52(1):87-92.

https://doi.org/10.3761/j.issn.0254-1769.2017.01.019.
